# Orientation‐Engineered Insulator‐Metal Transition in Vanadium Dioxide

**DOI:** 10.1002/advs.77134

**Published:** 2026-08-11

**Authors:** Xuanchi Zhou, Xiaohui Yao, Wentian Lu, Chunwei Yao, Xiaomei Qiao

**Affiliations:** ^1^ Key Laboratory of Magnetic Molecules and Magnetic Information Materials of Ministry of Education and School of Materials Science and Engineering Shanxi Normal University Taiyuan China; ^2^ Research Institute of Materials Science Shanxi Key Laboratory of Advanced Magnetic Materials and Devices Shanxi Normal University Taiyuan China

**Keywords:** correlated oxides, crystallographic orientation, insulator‐metal transition, protonation, vanadium dioxide

## Abstract

The artificial design of material microstructure in correlated oxides offers an emerging pathway for unlocking exotic electronic states through adjusting coupled order parameters. Here, we showcase the robust capability of a crystallographic orientation strategy in rationally designing insulator‐metal transition (IMT) functionality in the VO_2_ system, as driven by either critical temperature or protonation, giving rise to *in‐plane* anisotropic transport behavior and kinetically accelerated phase transition. Rutile‐on‐rutile epitaxy in VO_2_/TiO_2_ heterostructure enables the facile control over the *c*_R_‐axis orientation of VO_2_ films through engineering crystallographic orientations, which aligns the *c*_R_‐axis out of plane and enhances the orbital hybridization to extensively reduce the *T*_IMT_. Introducing a previously unexplored high‐Miller‐index (102) orientation offers an additional handle to tailor IMT behaviors in VO_2_ via mirror‐symmetry breaking, engendering *in‐plane* anisotropic IMT behaviors. Benefiting from tunable migration kinetics, hydrogen‐related electronic phase modulations in VO_2_/TiO_2_ (102) bilayer can be facilitated through inclined oxygen channels, a critical enabler for high‐speed iontronics. Hydrogen‐associated electronic orbital reconfigurations govern electronic localization of VO_2_ through protonation, as uncovered by theoretical calculations and synchrotron analysis. The present work identifies crystallographic orientation as a powerful tuning knob for adjusting IMT functionality in correlated systems, accessing exotic correlated electronic states.

## Graphical abstract

Crystallographic orientation engineering in VO_2_/TiO_2_ heterostructures enables tunable insulator‐metal transitions. The high‐Miller‐index (102) orientation breaks *in‐plane* mirror symmetry, inducing anisotropic transport, while its inclined oxygen channels accelerate protonation kinetics. This work establishes orientation design as a powerful knob for accessing exotic correlated electronic states and advancing high‐speed iontronic devices.

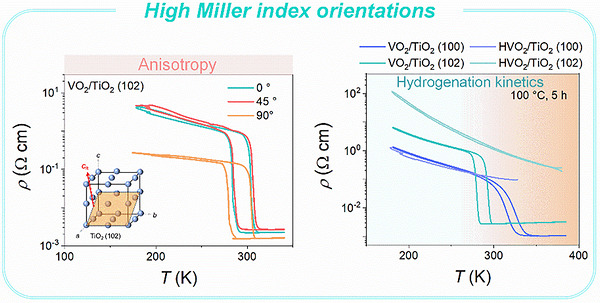

## 1 Introduction

The artificial design of material microstructure in correlated systems, ranging from superlattices and heterointerfaces to symmetry engineering, has opened a new frontier for unlocking emergent quantum states and physical phenomena [1, 2, 3, 4]. Transcending monolayer materials, controllable symmetry design, interfacial coupling, and geometry configuration in oxide heterostructures, achieved through epitaxial templates, drive anisotropic magnetoelectric responses in correlated systems, providing a fertile ground for designing emergent magnetoelectric states [5, 6]. This distinct advantage dictates controllable spatial arrangement of charge, spin, and orbital orders, fostering exotic quantum states such as two‐dimensional electron gas [7], interfacial ferromagnetic ordering [8, 9], and topological spin texture [10, 11]. In particular, the atomic‐scale manipulation of octahedral rotations and tilt in oxide lattices allows for precise control over the physical functionality. Specifically, on‐demand design of the RuO_6_ octahedral rotations in SrRuO_3_ system leads to the deterministic switching of perpendicular magnetization via breaking spatial inversion symmetry [12, 13, 14], while the high tolerance in NiO_6_ octahedral distortions engenders a tunable insulator‐metal transition (IMT) in *Re*NiO_3_ [15]. In particular, the ability to dynamically manipulate correlated magnetoelectric states through artificial microstructure design represents a paradigm shift, moving beyond native materials toward on‐demand quantum functionality. Notable examples include VO_2_ and SrFeO_2.5_, which exemplify the critical role of microstructure design in tailoring magnetoelectric transport behaviors via leveraging adjustable ionic evolution kinetics [16, 17, 18, 19].

Correlated VO_2_ system undergoes an abrupt first‐order IMT behavior from localized to itinerant electronic states at ∼340 K, determined by intertwined Peierls–Mott physics [20, 21]. Accompanied by an electronic phase transition, low‐symmetry monoclinic insulating VO_2_, featured by V–V dimerization, transitions to a rutile‐like structure with uniform V–V bonds [22, 23]. In this electron‐correlated system, the electron‐lattice coupling allows for facile control over the IMT functionality via artificial microstructure design, where the orbital hybridization between V‐3*d* and O‐2*p* orbitals is governed by the V–V chains along the *c*‐axis of rutile VO_2_ (*c*_R_) [24]. Typically, interfacial strain engineering provides a powerful tuning knob for adjusting the transition temperature (*T*_IMT_) in VO_2_ system, which readily decreases with a strengthened orbital hybridization via imparting compressive strain along the *c*_R_‐axis direction [25, 26]. To date, the epitaxial growth of VO_2_ films typically relies on low‐Miller‐index single‐crystalline templates with hexagonal or rutile structures, covering TiO_2_ and Al_2_O_3_ substrates [27, 28]. However, orbital hybridization in correlated oxide systems is fundamentally determined by crystal symmetry, introducing an additional freedom to design the IMT property of VO_2_. Consequently, extending to crystal planes with high index orientation offers a feasible route to deliberately lower *in‐plane* mirror symmetries in correlated VO_2_, accessing novel functionalities unobtainable in the traditional phase diagram.

In this work, we realize the precise control over IMT behaviors in a correlated VO_2_ system, as driven by critical temperature or hydrogen doping, through artificially engineering the crystallographic orientation. We design a series of VO_2_/TiO_2_ heterostructures with diverse crystallographic orientations, among which a novel high‐index (102) orientation is introduced, resulting in tunable IMT functionalities via adjusting interfacial strain states along the *c*_R_‐axis. Crystallographic orientation design that aligns the *c*_R_‐axis out of plane markedly enhances V‐3*d* and O‐2*p* orbital hybridization in VO_2_ through bandwidth control, reducing resultant *T*_IMT_. Such the high‐index (102) orientation gives rise to *in‐plane* anisotropic temperature‐controlled IMT behaviors in the VO_2_ system, while facilitating hydrogen‐triggered electronic phase modulations leveraging an inclined oxygen channel. Our present work showcases the robust capability of artificial crystallographic orientation design in unlocking exotic electronic states in correlated oxide systems exhibiting the IMT.

## 2 Results and Discussion

### 2.1 The Artificial Design of Crystallographic Orientation in VO_2_ System

**FIGURE 1:**
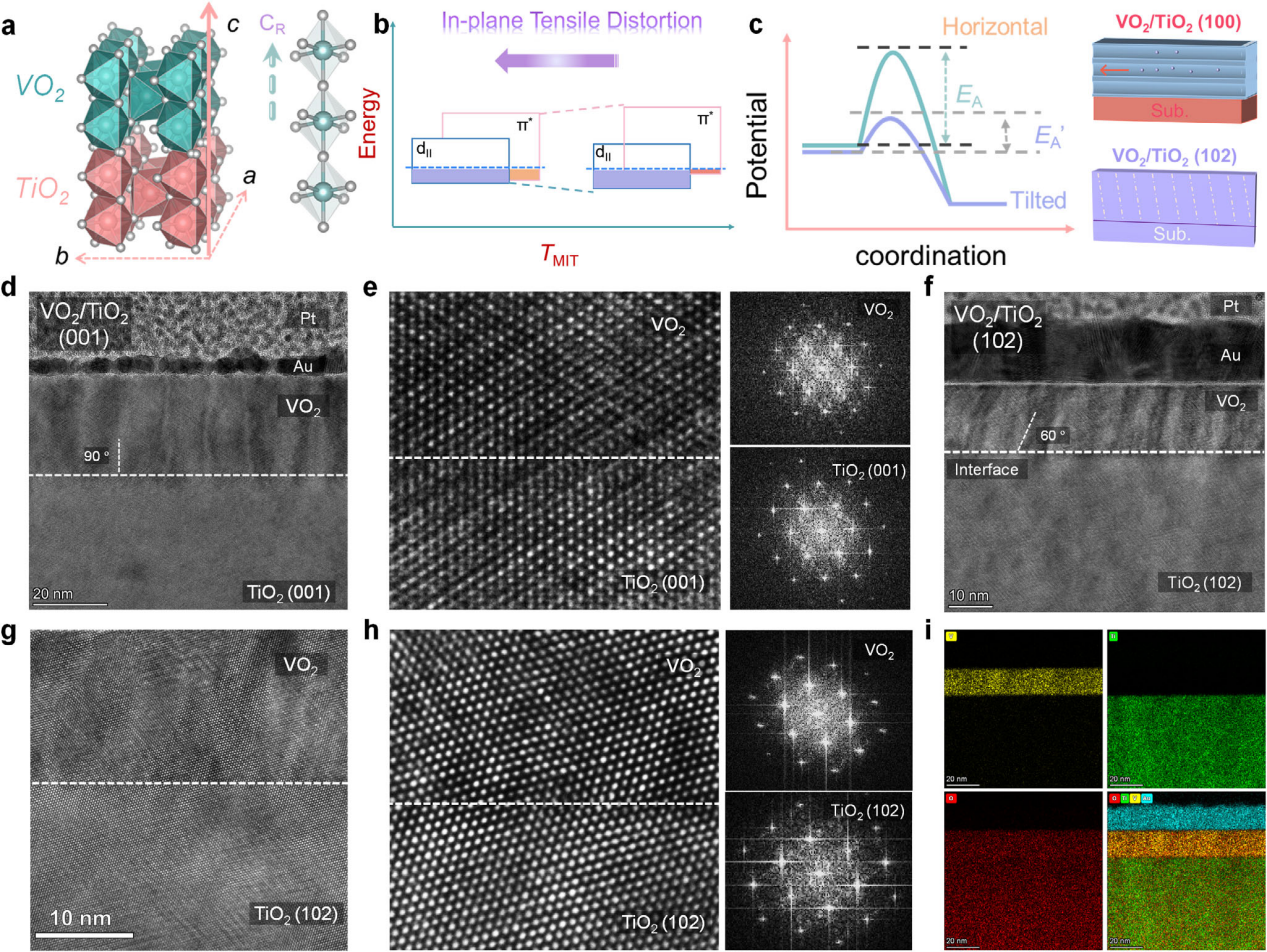
Realization of crystallographic orientation design in correlated VO_2_. (a) Schematic diagram of as‐deposited VO_2_/TiO_2_ heterostructure and *c*_R_‐axis direction. (b) Schematic of tailoring the electronic phase transitions of VO_2_ via band‐filling control using interfacial strains. (c) Schematic of ionic diffusion barriers in oxide lattice via microstructure design. (d) High‐resolution transmission electron microscopy (HRTEM) images for VO_2_/TiO_2_ (001) heterostructure. (e) Zoom‐in images for cross‐sectional HRTEM and respective Fast Fourier Transform (FFT) images for VO_2_/TiO_2_ (001) heterostructure. (f,g) HRTEM images for VO_2_/TiO_2_ (102) heterostructure. (h) Zoom‐in images for cross‐sectional HRTEM and respective FFT images for VO_2_/TiO_2_ (102) heterostructure. (i) Vanadium, titanium and oxygen elementary distributions for as‐grown VO_2_/TiO_2_ heterostructure characterized by using energy dispersion spectrum (EDS).

Correlated VO_2_ system, featured by an intimate electron‐lattice coupling, offers an ideal testbed for designing correlated electronic states through the artificial design of crystallographic orientations. The IMT in the VO_2_ system, controlled by critically balanced Peierls‐Mott physics, can be effectively adjusted through bandwidth control, in which the V–V chains oriented parallel to the *c*_R_‐axis determine the V‐3*d* and O‐2*p* orbital hybridization (Figure 1a). Imparting a compressive distortion along the *c*_R_‐axis of VO_2_ enhances the orbital hybridization between V‐3*d* and O‐2*p* orbitals to reduce the resulting *T*_IMT_ via strengthening relative stability in metallic orbital configuration, providing a powerful handle to design IMT property (Figure 1b) [26]. Moreover, introducing inclined aligned domain boundaries readily lowers the energy barrier for ionic migration in the oxide lattice in comparison with the horizontally aligned ones, a critical factor for developing high‐speed iontronic devices (Figure 1c). To address this central concept, taking the VO_2_/TiO_2_ heterostructure as a model system, crystallographic orientation engineering is exploited to adjust the IMT functionality of VO_2_. Benefiting from a similar *a*‐axis lattice constant between the VO_2_ film (*a*_0, film_ = 4.5546 Å) and TiO_2_ substrate (*a*_0, sub._ = 4.5937 Å) and identical crystalline structure, the rutile‐on‐rutile epitaxy allows for flexible control over the crystallographic orientations of the grown VO_2_ films. This expectation is further validated by respective high‐resolution transmission electron microscopy (HRTEM) in VO_2_/TiO_2_ (001) heterostructure, which clearly reveals direct rutile‐on‐rutile epitaxial growth of VO_2_ film deposited on *c*‐plane TiO_2_ substrate, with vertically aligned V–V chains resolved (Figure 1d,e). Given that the V–V chains are parallel with the *c*_R_‐axis, (001)‐oriented TiO_2_ substrate induces a *c*‐plane preferential orientation in the grown VO_2_ films.

In order to effectively adjust the *c*_R_‐axis orientation, a high Miller index (102)‐oriented TiO_2_ substrate is employed to deposited VO_2_ films, as their HRTEM images shown in Figure 1f–h and Figure S1. An inclined V–V chain with a tilt angle of ∼60°is visualized in the as‐deposited VO_2_/TiO_2_ (102) heterostructure, where expected rutile structures are identified. Despite the adjacent atomic numbers of vanadium (*Z* = 23) and titanium (*Z* = 22) element hinder the precise spatial resolution of the heterointerface, energy dispersion spectrum (EDS) provides compelling evidence for elementary distribution in the VO_2_/TiO_2_ (102) bilayer. A chemically distinct heterointerface is observed for the as‐deposited VO_2_/TiO_2_ (102) system, without pronounced elemental intermixing (Figure 1i). In addition, the film thickness is estimated to be around 15 and 20 nm for VO_2_/TiO_2_ (102) and VO_2_/TiO_2_ (001) heterostructures using TEM and atomic force microscope (AFM) techniques, respectively (Figures S2 and S3), with AFM also confirming relatively smooth film surfaces (Figure S4). The realization of controllable crystallographic orientations in VO_2_ films through engineering the substrate orientations offers a promising platform for adjusting the IMT property of VO_2_.

### 2.2 Manipulating Thermally‐Driven IMT Through Crystallographic Orientation

**FIGURE 2:**
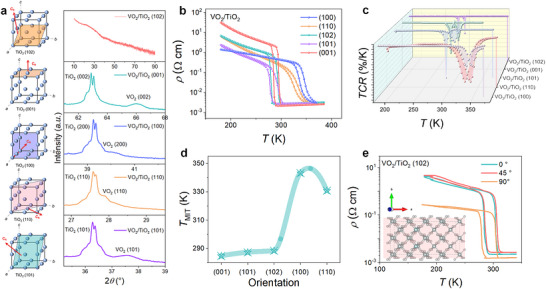
Manipulating the IMT property of VO_2_ via using crystallographic orientation. (a) Schematic illustration of X‐ray diffraction (XRD) patterns and *c*_R_‐axis orientation of VO_2_ grown on TiO_2_ substrates with different orientations. (b) Temperature dependence of material resistivity (*ρ*‐*T*) as compared for VO_2_/TiO_2_ heterostructure with different orientations. (c) Temperature coefficient of material resistivity compared for VO_2_/TiO_2_ heterostructure with different orientations. (d) Orientation‐dependent transition temperature (*T*_MIT_) of VO_2_/TiO_2_ heterostructure. (e) *ρ*‐*T* as compared for VO_2_/TiO_2_ (102) heterostructure along different *in‐plane* directions.

Rutile‐on‐rutile epitaxial growth in VO_2_/TiO_2_ heterostructures enables precise control over the *c*_R_‐axis orientation of VO_2_ films through engineering crystallographic orientation of TiO_2_ substrates. Consequently, we introduce a series of crystallographic orientations for single‐crystalline TiO_2_ substrates, covering low‐Miller‐index (001), (100), (110), and (101) orientations and high‐Miller‐index (102) orientation. The preferential growth of the grown VO_2_ films on differently oriented TiO_2_ substrates is demonstrated by respective X‐ray diffraction (XRD) patterns (Figure S5), whereas no discernable diffraction peaks are observed from the (102)‐oriented TiO_2_ substrate due to systematic extinction (Figure 2a). Notably, (001)‐, (102)‐, and (101)‐oriented TiO_2_ substrates induce the *c*_R_‐axis of VO_2_ to align *out‐of‐plane*, subjecting it to a compressive strain, albeit with different tilt angles. In contrast, (100)‐ and (110)‐oriented counterparts drive the *c*_R_‐axis into an *in‐plane* alignment, theoretically resulting in tensile strain states along the *c*_R_‐axis direction. Engineering the *c*_R_‐axis to align *out‐of‐plane* significantly lowers the required growth temperature of VO_2_ films from 400°C to 260°C (Figure S6), possibly attributed to a reduced nucleation barrier that stems from more favorable interfacial lattice matching along this orientation [29]. In addition, for VO_2_ films deposited on TiO_2_ (001) at temperatures above 300°C, the possible intermixing and Ti interdiffusion into the upper VO_2_ layer can result in a stepwise IMT behavior that deviates from the expected *T*_IMT_ (Figure S7). Furthermore, the possibly higher oxygen vacancy concentration in VO_2_ films deposited at a higher temperature may also affect the resultant IMT property.

Imparting a compressive (tensile) distortion along the *c*_R_‐axis of VO_2_ is expected to enhance (depress) the V‐3*d* and O‐2*p* orbital hybridization, reducing (elevating) the resultant *T*_IMT_. This interpretation is validated by respective temperature dependences of the resistivity (*ρ‐T*) for VO_2_ films deposited on differently oriented TiO_2_ substrates (Figure 2b). It is found that as‐grown VO_2_ films undergo abrupt IMT behaviors, with resistive changes exceeding 2–3 orders of magnitude across the critical point of *T*_IMT_. The *T*_IMT_ for VO_2_ films is determined by averaging the temperatures at which the absolute value of the temperature coefficient of resistance (*TCR*) reaches its maximum (Figure 2c). Of particular note is the tunable *T*_IMT_ in VO_2_ films achieved through crystallographic orientation engineering (Figure 2d), wherein an extensively reduced *T*_IMT_ in the range of 284.73–288.47 K is realized when the *c*_R_‐axis is aligned out of plane (e.g., (001)‐, (102)‐, and (101)‐orientations). The compressive strain along the *c*_R_‐axis of VO_2_, resulting from the epitaxial TiO_2_ template, strengthens the V‐3*d* and O‐2*p* orbital hybridization, thereby reducing the *T*_IMT_. Surprisingly, the reduction in *T*_IMT_ does not scale linearly with the tilt angle of the *c*_R_‐axis relative to the *c*‐axis. This nonlinearity reflects that, in correlated systems, bandwidth modulation can induce an abrupt change in orbital hybridization once the tilt angle of the *c*_R_ axis relative to the *c* axis decreases below a critical value of ∼60°, rather than a rigid response to interfacial strain states (Figure S8). In addition, the intertwined Mott physics associated with strong electron correlations further contributes to such nonlinear behavior. It is worthy to note that elevating the film thickness of the VO_2_ film readily alleviates the role of interfacial strains in engineering the IMT property, but this behavior is distinct from that of orientation engineering (Figures S9 and S10). By strong contrast, an *in‐plane* alignment of the *c*_R_‐axis elevates the *T*_IMT_ of VO_2_, as exemplified by the (100)‐faceted VO_2_ film (e.g., 343.19 K). Nevertheless, manipulating the *T*_IMT_ of VO_2_ via tensile strain along the *in‐plane c*_R_‐axis is restricted by a relatively high lattice mismatch (∼2.85%) between the VO_2_ (*c*_0, film_ = 2.876 Å) and TiO_2_ (*a*_0, sub._ = 2.958 Å). In contrast to depositing on TiO_2_ substrates with low Miller index (Figure S11), forming the interfaces on crystal planes of high Miller index (102) results in the breaking of the *in‐plane* mirror symmetry and gives rise to *in‐plane* anisotropic IMT transport behaviors (Figure 2e) [5]. Nevertheless, no pronounced anisotropy in *in‐plane* electrical transport is detected for the VO_2_ film deposited on low‐Miller‐index (100) TiO_2_ substrate (Figure S12), which further clarifies the role of symmetry design through high‐Miller‐index crystallographic orientation in engineering the IMT property. This finding unravels that introducing high‐Miller‐index substrate readily engenders a low‐symmetry system, offering a feasible route to artificially establish anisotropic transportation behaviors in correlated systems.

### 2.3 Tunable Protonation Kinetics Via Using Crystallographic Orientation Design

**FIGURE 3:**
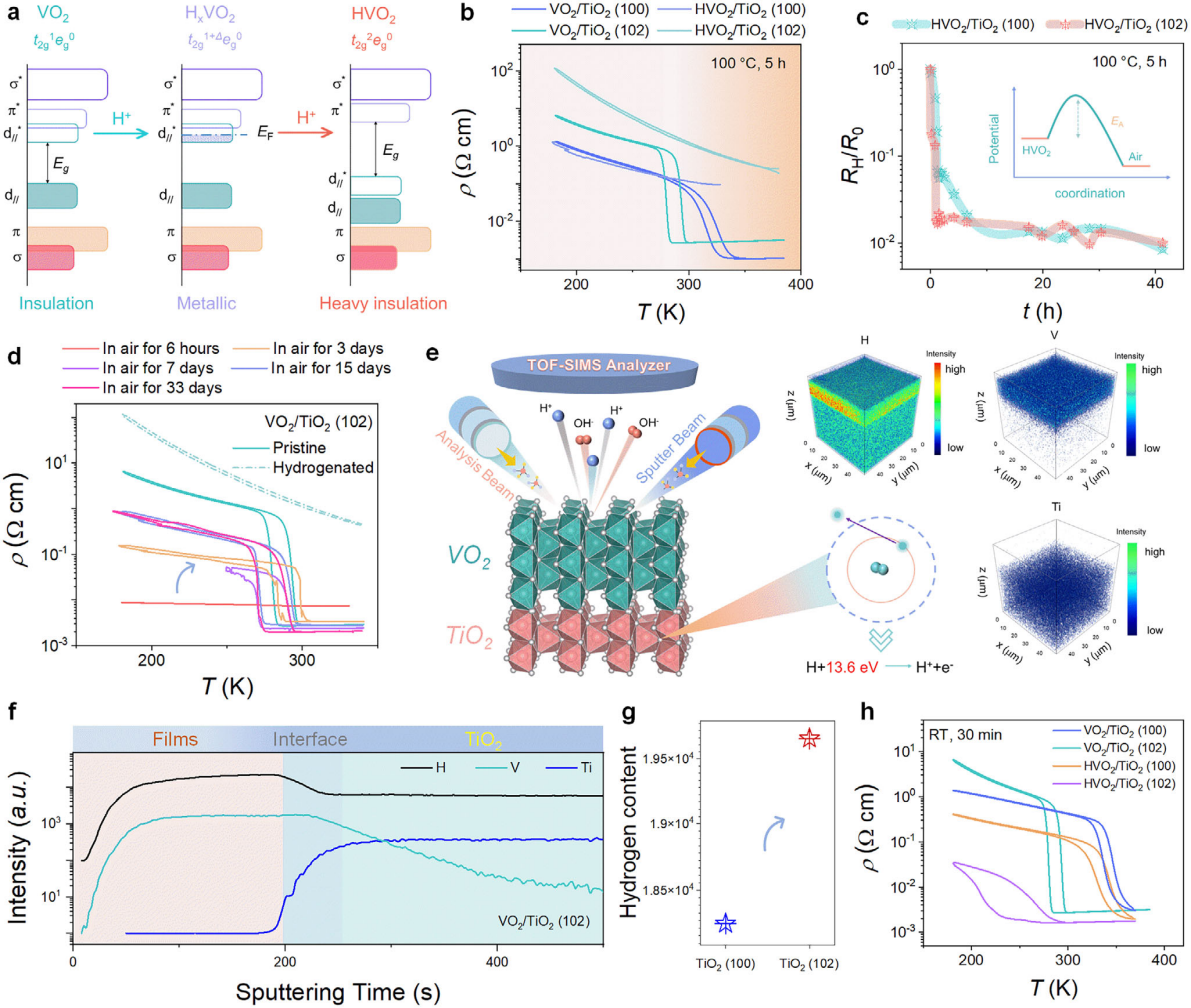
Adjustable protonation kinetics in correlated VO_2_ system. (a) Schematic of hydrogen‐triggered electronic orbital reconfiguration in correlated VO_2_ system. (b) *ρ*‐*T* curves compared for VO_2_/TiO_2_ heterostructure through hydrogenation. (c) Dehydrogenation process as compared for VO_2_/TiO_2_ heterostructure via exposing to the air. (d) *ρ*‐*T* curves compared for VO_2_/TiO_2_ (102) heterostructure via exposing to the air. (e) Schematic of the principle in time‐of‐flight secondary ion mass spectrometry (ToF‐SIMS) and three‐dimensional ToF‐SIMS element maps for VO_2_/TiO_2_ heterostructure. (f) Depth profile of the hydrogen, titanium and vanadium element distribution within the lattice of VO_2_ film deposited on the TiO_2_ (102) substrate. (g) Comparison of hydrogen content at domain boundaries with different tilt angles. (h) *ρ*‐*T* as measured for VO_2_/TiO_2_ heterostructure as hydrogenated at room temperature for 30 min.

Apart from adjustable thermally‐driven IMT with *in‐plane* anisotropy, the design of crystallographic orientation provides a testbed for tailoring hydrogen‐related electronic phase modulations via susceptional protonation kinetics [30]. Transcending conventional IMT driven by a critical temperature, the incorporation of mobile hydrogens triggers isothermal electronic phase transitions in the VO_2_ system along the insulator‐metal‐highly insulator pathway [31]. The partial occupation in the low‐energy *d*_//_^*^ orbital of VO_2_ through hydrogen doping drives an electron delocalization, while the excessive hydrogenation results in the complete electron filling in *d*_//_^*^ orbital, instead triggering electron localization (Figure 3a) [32]. To effectively introduce the hydrogen doping into the VO_2_ lattice, a Pt‐assisted hydrogen spillover strategy is herein employed, where the sputtered Pt dots as a catalyst extensively reduce the energy barrier for dissociating H_2_ molecule at the triple phase boundary (Figure S13) [33, 34]. Analogous to prior reports, low‐temperature protonation for a prolonged period (e.g., 100°C, 5 h) leads to an electron‐localized hydrogenated phase of VO_2_, regardless of crystal facet orientation, as evidenced by their *ρ‐T* tendencies in Figure 3b. The protonation of VO_2_ at lower temperature (e.g., 100°C) offers a feasible pathway to avoid the potential impacts of oxygen deficiency in IMT. Although both (100)_R_‐ and (102)_R_‐faceted VO_2_ exhibit typical insulating transport behavior upon hydrogenation, hydrogen‐triggered resistive modulation (e.g., *R*_H_/*R*_0_) in (102)_R_‐faceted VO_2_ exceeds that of (100)_R_‐faceted counterpart under identical protonation conditions by 2 orders of magnitude (Figure S14). This finding aligns well with the carrier activation energy (*E*_a_) derived from fitting *ρ‐T* tendencies: protonation leaves the *E*_a_ of (100)_R_‐faceted VO_2_ largely unchanged, whereas it doubles that of (102)_R_‐faceted counterpart (Figure S15). These results provide evidence for accelerated protonation kinetics in VO_2_/TiO_2_ system through the artificial design of crystallographic orientation, which is critical for the development of high‐speed iontronic devices.

Further consistency in the crystallographic orientation‐dependent manipulation of proton migration in VO_2_ is demonstrated by the respective dehydrogenation kinetics shown in Figure 3c. The ultrahigh mobility of protons with the smallest ionic radius enables reversible hydrogen‐triggered phase transitions in VO_2_, where the hydrogen‐driven electron‐localized state readily reverts to the pristine state upon exposure to ambient atmosphere. Similarly, the (102)_R_‐faceted VO_2_ exhibits a shorter onset time for the significant reduction in resistivity compared to its (100)_R_‐faceted counterpart. Furthermore, the VO_2_/TiO_2_ (102) heterostructure recovers its IMT property after three days in ambient atmosphere, albeit with incomplete restoration due to residual protons in the deeper layer (Figure 3d). The reversibility in the ionic control over electronic state evolution of VO_2_ benefits iontronic device applications. It is worth noting that an electron‐localized state of (102)‐faceted VO_2_ initially transits to a transient state with a reduced material resistivity, and eventually recovers directly to the thermodynamically stable pristine state, rather than to a hydrogenated electron‐itinerant state.

To clarify the role of hydrogen in electronic state evolution of VO_2_ system, time‐of‐flight secondary ion mass spectrometry (ToF‐SIMS) analysis is performed, in which an ion beam (e.g., Cs^+^) sputters the VO_2_ heterostructure and the intensity of ejected molecular ions over sputtering time reveals the depth profile of incorporated hydrogen [35]. The effective incorporation of hydrogen is evidenced by three‐dimensional ToF‐SIMS element maps for hydrogenated VO_2_/TiO_2_ (102) heterostructure shown in Figure 3e, where a more pronounced H signal is observed for the VO_2_ film region in comparison with the TiO_2_ substrate. This result is consistent with the depth profile of elementary distribution in VO_2_/TiO_2_ (102) bilayer, in which the presence of hydrogen is evident over the film region via hydrogenation (Figure 3f). Three‐dimensional element maps and the corresponding depth profile of elementary distribution for VO_2_/TiO_2_ (100) bilayer through hydrogenation are summarized in Figures S16 and S17. In particular, the achievable hydrogen concentration in VO_2_/TiO_2_ (102) bilayer is slightly higher than that of (100)_R_‐oriented counterpart (Figure 3g). Given similarly expedited dehydrogenation kinetics, no pronounced discrepancy in incorporated hydrogen concentration can be discerned within the VO_2_ lattice. Such kinetically accelerated (de)hydrogenation kinetics through crystallographic orientation design is further demonstrated by a relatively mild hydrogenation (e.g., room temperature, 30 min) (Figure 3h), consistent with the protonation at 70°C for 30 min (Figure S18). Specifically, a mild protonation drives a significant reduction in the *T*_IMT_ for (102)_R_‐faceted VO_2_, with a dramatic suppression in the transition sharpness, contrasting sharply with (100)_R_‐oriented counterpart, where an abrupt IMT is still persisted. Protons preferentially migrate along empty oxygen channels; thus, inclined or vertical channels with open ends at the VO_2_ surface promote oxygen ionic migration more efficiently than their horizontally aligned counterparts [19]. Consequently, crystallographic orientation design in VO_2_ system allows for an active manipulation of phase transitions in correlated oxides, induced either by critical temperature or by hydrogen incorporation.

### 2.4 Physical Origin Behind Hydrogen‐Related Electronic Phase Modulations

**FIGURE 4:**
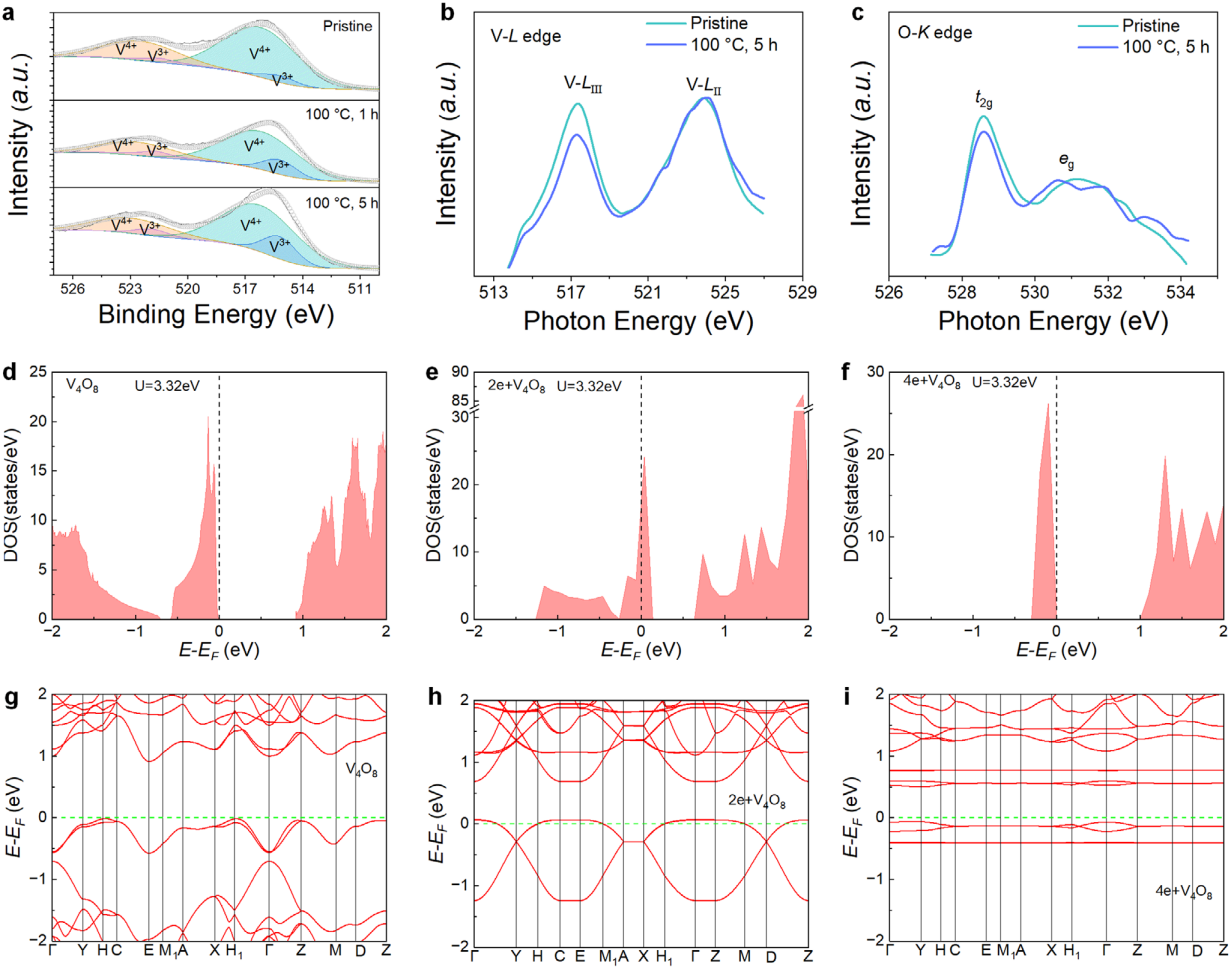
Band structure regulation in VO_2_ through hydrogenation. (a) X‐ray photoelectron spectra (XPS) spectra for the core levels of vanadium of hydrogenated VO_2_/TiO_2_ (102) heterostructure. (b,c) Soft X‐ray absorption spectroscopy (sXAS) for the V‐*L* edge and O‐*K* edge of VO_2_/TiO_2_ heterostructure through hydrogenation. (d–f) Calculated density of states (DOS) of VO_2_ film before and after hydrogenation. (g–i) Calculated fat band structure for pristine VO_2_, and hydrogenated VO_2_.

To probe the physical origin governing hydrogen‐related electronic phase modulations in VO_2_ system, X‐ray photoelectron spectra (XPS) and soft X‐ray absorption spectroscopy (sXAS) techniques are conducted, as the V 2*p* core‐level XPS spectrum shown in Figure 4a and Figure S19. Performing the hydrogenation of (102)_R_‐oriented VO_2_ at 100°C gives rise to the reduction in the valence state of vanadium from V^4+^ to V^(4‐δ)+^, due to hydrogen‐related electron doping. In addition, the V‐*L* edge spectrum in sXAS spectra, resulting from the V 2*p* → 3*d* transition, is recognized as an effective indicator for probing the changes in vanadium valence state (Figure 4b). The leftward shift of both V‐*L*_III_ and V‐*L*_II_ peaks is observed for VO_2_/TiO_2_ (102) bilayer through hydrogenation, in agreement with XPS result. Considering empty O‐2*p* states and intimate orbital hybridization between V 3*d* and O 2*p* orbitals, the relative variation in the O‐*K* edge peak intensity can reflect the electron occupancy in V‐3*d* orbital [36]. Protonation leads to a reduction in the first peak intensity relative to the second peak, unveiling the electron filling in the low‐energy occupation in the *t*_2g_ band of VO_2_ (Figure 4c).

The electronic band structure is further analyzed using density functional theory (DFT) calculations, with the evolution of the band structure upon protonation shown in Figure 4d–i. It can be seen that a band gap of ∼0.8 eV is observed for pristine VO_2_, featured by an electron‐correlated ground state (Figure 4d), aligning well with the calculated electronic band structure (Figure 4g). Introducing two electron carriers into unoccupied conduction band states results in the metallization of VO_2_, as exemplified by finite DOS near the *E*_F_ (Figure 4e). However, the integer filling via the donation of four electrons into VO_2_ instead opens a new band gap (Figure 4f–i), demonstrating the emergence of an electron‐localized state. Therefore, multi‐state electronic phase modulations realized in VO_2_ through hydrogenation, followed by insulator (*t*_2g_^1^ *e*_g_^0^)‐metal (*t*_2g_^1+Δ^ *e*_g_^0^)‐highly insulator (*t*_2g_^2^ *e*_g_^0^) pathway, are determined by hydrogen‐associated band‐filling control over electronic band structure.

## 3 Conclusions

In summary, we present a promising crystallographic orientation strategy to design the IMT functionality in a correlated VO_2_ system, as triggered by critical temperature or protonation, unlocking exotic electronic states. Harnessing the rutile‐on‐rutile epitaxy in VO_2_/TiO_2_ heterostructure, the flexible control over the *c*_R_‐axis direction in VO_2_ system, achieved through engineering the substrate orientations, results in tunable thermally‐driven IMT properties via bandwidth control. Introducing a high‐Miller‐index (102) orientation breaks the mirror symmetry of the grown VO_2_ film, giving rise to *in‐plane* anisotropic IMT behaviors. From the perspective of ionic evolution, an inclined oxygen channel realized in VO_2_/TiO_2_ (102) heterostructure kinetically facilitates protonation kinetics, in comparison with the horizontally aligned ones, a critical enabler for high‐speed protonic devices. Utilizing the sXAS analysis and DFT calculations, we clarify the hydrogen‐related filling‐controlled electronic phase transitions in VO_2_ that are governed by electronic orbital reconfiguration. Our present findings provide a new guideline for rationally designing exotic electronic states and IMT functionality in correlated oxide systems through crystallographic orientation design.

## 4 Experimental Section

### 4.1 Fabrication of As‐Deposited VO_2_/TiO_2_ Heterostructures

VO_2_ films were deposited on single‐crystalline TiO_2_ substrates with different crystallographic orientations, including (001), (100), (110), (101), and (102), using the laser molecular beam epitaxy (LMBE). The oxygen pressure, target‐substrate distance, and laser fluence were fixed at 2.0 Pa, 45 mm, and 1.0 J cm^−2^, respectively, while the growth temperature was tailored to each orientation. Specifically, (100)‐ and (110)‐oriented VO_2_ films were grown at 400°C, (101)‐ and (102)‐oriented films at 275°C, and (001)‐oriented films at 260°C. After deposition, the samples were cooled to room temperature under the same oxygen partial pressure. Prior to hydrogenation, platinum dots of approximately 20 nm thickness were sputtered onto the surface of the as‐deposited VO_2_/TiO_2_ heterostructures. Finally, following the hydrogen spillover strategy, the VO_2_/TiO_2_ heterostructures were annealed in an H_2_/Ar gas mixture to achieve effective hydrogen incorporation.

### 4.2 Material Characterizations

The crystal structures of the grown VO_2_ films were probed by X‐ray diffraction (XRD) (Rigaku, Ultima IV). High‐resolution transmission electron microscopy (HRTEM) (JEOL, JEM F200; JEM, ARM300F) was also performed to characterize the crystal structure, for which the VO_2_ films were first prepared using focused ion beam (FIB) (FEI, G4 UX). Variations in the chemical environment of hydrogenated VO_2_ were assessed by X‐ray photoelectron spectroscopy (XPS) (Thermo, K‐Alpha X). The electronic structure of the VO_2_ films was further investigated through soft X‐ray absorption spectroscopy (sXAS) at beamline BL08U1A of the Shanghai Synchrotron Radiation Facility (SSRF). The raw XAS data were first differentiated to separate the V *L*‐edge (510–527.5 eV) and O *K*‐edge (527.5–538.7 eV) regions; the V *L*‐edge spectra were then normalized to unity in the 0–1 range after background subtraction, while the O *K*‐edge spectra were normalized to the intensity of the *e*_g_ peak (second peak in O *K*‐edge spectra) to emphasize the changes in *t*_2g_/*e*_g_ peak intensity. Additionally, all XAS spectra were acquired in total electron yield (TEY) mode. Temperature‐dependent resistivity of the deposited VO_2_ films was measured using a Physical Property Measurement System (PPMS, Quantum Design), while room‐temperature resistance was recorded with a Keithley 2400 system. The depth profile of hydrogen was examined by time‐of‐flight secondary ion mass spectrometry (ToF‐SIMS) technique (ION‐TOF GmbH, TOF.SIMS 5).

### 4.3 First‐Principles Calculations

First‐principles calculations were performed using the projector augmented wave (PAW) method using the QUANTUM ESPRESSO package. The plane‐wave kinetic energy cutoff was set to 90 Ry, and the Brillouin zone was sampled by using a 5 × 11 × 9 Monkhorst‐Pack k‐point mesh. Electron correlations in the VO_2_ system were realized by using a GGA+U method [37], applying a Hubbard *U* of 3.32 eV to the V‐3*d* states. Structural relaxation proceeded until the interatomic forces fell below 10^−3^ Ry/Bohr. Self‐consistent field calculations converged to an energy criterion of 10^−12^ Ry, which ensures the high numerical accuracy.

## Author Contributions

X.Z. conceived this study and led the project; X.Z. planned the experiment and analyzed the results; X.Y. and X.Q. grew VO_2_ films and carried out the transport measurements under the supervision of X.Z.; W.L. performed the DFT calculations assisted by C.Y.; X.Z. wrote the paper with contributions from all authors; All authors discussed the results and commented on the final manuscript.

## Conflicts of Interest

The authors declare no conflict of interest.

## Supporting information




**Supporting File**: advs77134‐sup‐0001‐SuppMat.docx.

## Data Availability

The data that support the findings of this study are available from the corresponding author upon reasonable request.
