# Supplementary material for: Orientation‐Engineered Insulator‐Metal Transition in Vanadium Dioxide
Source: Adv Sci (Weinh). 2026 Aug 11:e77134. Online ahead of print. doi: 10.1002/advs.77134 (PMC13460274; doi:10.1002/advs.77134)
Supplement: Supplementary file 1 [file ADVS-9999-e77134-s001.docx]

Supporting Information

**Orientation‑engineered insulator-metal transition in vanadium dioxide**

*Xuanchi Zhou* *^1, 2 *^,* *Xiaohui Yao ^1^, Wentian Lu ^1, 2^, Chunwei Yao ^1^, Xiaomei Qiao ^1^*

*^1^* *Key Laboratory of Magnetic Molecules and Magnetic Information Materials of Ministry of Education & School of Materials Science and Engineering, Shanxi Normal University, Taiyuan,* *030031, China*

*^2^ Research Institute of Materials Science, Shanxi Key Laboratory of Advanced Magnetic Materials and Devices, Shanxi Normal University, Taiyuan 030031, China*

*Authors to whom correspondence should be addressed: [*xuanchizhou@sxnu.edu.cn*](mailto:xuanchizhou@sxnu.edu.cn) *(X. Zhou)*.

**This file includes:**

Supplementary Figures 1-19 and Supplementary References.

**Contents**

**Figure S1. TEM images for as-deposited VO_2_/TiO_2_ (102) heterostructure.**

**Figure S2. HRTEM images for VO_2_/TiO_2_ (102) and VO_2_/TiO_2_ (001) heterostructure.**

**Figure S3. The film thicknesses of VO_2_/TiO_2_ as measured by using AFM technology.**

**Figure S4. AFM images for VO_2_/TiO_2_ with different orientations.**

**Figure S5. Full spectra for XRD patterns of VO_2_ films grown on TiO_2_ substrates with different orientations.**

**Figure S6. Growth temperature for VO_2_ deposited on TiO_2_ substrates with different orientations.**

**Figure S7. The *ρ*-*T* curves as compared for VO_2_/TiO_2_ (001) deposited on different temperatures.**

**Figure S8. The relationship between the *T*_IMT_ and the tilt angle (**$\boldsymbol{\theta}$**) of the *c*_R_ axis relative to the *c* axis.**

**Figure S9.** **Comparing the role of orientation design and film thickness in engineering the IMT property of VO_2_.**

**Figure S10.** **The *ρ*-*T* curves as compared for VO_2_/TiO_2_ (102) with different thickness.**

**Figure S11. Minor symmetry of VO_2_ deposited on low-Miller-index substrates.**

**Figure S12. The *ρ*-*T* curves as compared for VO_2_/TiO_2_ (100) heterostructure along different *in-plane* directions.**

**Figure S13. Schematic illustration of the hydrogen spillover method.**

**Figure S14. Hydrogenation-induced resistance modulation.**

**Figure S15. Comparison of *E*_a_ for VO_2_/TiO_2_ heterostructure upon hydrogenation.**

**Figure S16. Three-dimensional element maps for hydrogenated VO_2_/TiO_2_ (100).**

**Figure S17. Depth profile of elementary distribution for hydrogenated VO_2_/TiO_2_ (100).**

**Figure S18. Reversibility in the proton evolution of VO_2_/TiO_2_ system.**

**Figure S19. Full spectra for sXAS of VO_2_/TiO_2_ heterostructure.**

**Supplementary References**

**Supplementary Figure 1.** **a-b**, Low-magnification and **c**, high-magnification cross-sectional transmission electron microscopy (TEM) images for as-deposited VO_2_/TiO_2_ (102) heterostructure. Tilted domain boundaries were observed for the VO_2_/TiO_2_ (102) heterostructure through rutile-on-rutile epitaxy induced by using TiO_2_ template, differing from the vertical boundaries in VO_2_/TiO_2_ (001). Therefore, the direct epitaxy growth of VO_2_ film on TiO_2_ substrates serves as a fertile ground for designing correlated electronic states in VO_2_ system via using crystallographic orientation strategy.^[1]^

**Supplementary Figure 2.** Cross-sectional transmission electron microscopy (TEM) images for **a**, VO_2_/TiO_2_ (102) and **b**, VO_2_/TiO_2_ (001) heterostructure. The film thicknesses of the as-deposited VO_2_/TiO_2_ (102) and VO_2_/TiO_2_ (001) heterostructures were determined by using TEM technique to be about 15 nm and 20 nm, respectively.

**Supplementary Figure 3.** The film thicknesses of VO_2_/TiO_2_ heterostructures as measured by using AFM technology. The film thickness of the (100)-, (110)- and (101)-oriented VO_2_/TiO_2_ heterostructure are approximately 20.3 nm, 20.6 nm and 21.3 nm, while the thicknesses of the (001)-oriented and (102)-oriented VO_2_/TiO_2_ heterostructure are 19.7 nm and 15.8 nm, respectively, which are consistent with the thicknesses observed by using TEM technique.

**Supplementary Figure 4.** Atomic force microscope (AFM) images for as-grown **a,** (102)-oriented, **b,** (001)-oriented and **c,** (100)-oriented VO_2_/TiO_2_ heterostructure. The atomically smooth film surfaces of as-grown VO_2_/TiO_2_ system are identified using AFM technique, characterized by the root-mean-square roughness (*R*_q_) values below 0.5 nm.

**Supplementary Figure 5.** Full spectra for X-ray diffraction (XRD) patterns of VO_2_ films grown on TiO_2_ substrates with different orientations. The preferential orientation growth of VO_2_ films deposited on differently oriented TiO_2_ substrates is identified through XRD patterns.

**Supplementary Figure 6. a,** The sample photographs of VO_2_/TiO_2_ heterostructures with various orientations deposited at substrate temperature of 275 °C. **b,** Comparison of temperature dependence of material resistivity (*ρ*-*T*) curves of the VO_2_/TiO_2_ (001) and VO_2_/TiO_2_ (102) heterostructure deposited at substrate temperature of 275 °C. **c,** Relationship between growth temperature and crystallographic orientation of TiO_2_ substrate. At a fixed growth temperature of 275 °C, brownish-yellow VO_2_ films were observed for the ones deposited on the TiO_2_ (101) and TiO_2_ (102) substrates, whereas no discernable VO_2_ film was detected on TiO_2_ (100) or TiO_2_ (110) substrates. When the growth temperature was elevated to 350 °C, brownish-yellow films formed on TiO_2_ (100) and TiO_2_ (110); however, *ρ*-*T* measurements revealed a semiconducting transport behavior without any signs of expected insulator-metal transition (IMT). It was not until the temperature was further increased to 400 °C that VO_2_ films exhibiting a pronounced IMT were successfully obtained on TiO_2_ (100) or TiO_2_ (110) substrates. Therefore, aligning the *c*_R_-axis out of plane can effectively reduce the growth temperature of VO_2_ through lattice matching, differing from the ones with the *c*_R_-axis orientation along the *in-plane* direction.

**Supplementary Figure 7.** The *ρ*-*T* curves as compared for VO_2_/TiO_2_ (001) deposited on different temperatures. The possible intermixing and Ti interdiffusion into the upper VO_2_ film deposited on (001)-oriented TiO_2_ can readily disrupt the IMT property of VO_2_ at higher deposition temperatures, resulting in a stepwise IMT behavior that deviates from the expected *T*_IMT_ at a deposition temperature of 400 °C.

**Supplementary Figure 8.** The relationship between the *T*_IMT_ and the tilt angle ($\theta$) of the *c*_R_ axis relative to the *c* axis. The orbital reconfiguration becomes abrupt once the tilt angle of the *c*_R_ axis relative to the *c* axis decreases below a critical value of ~60 º, reflecting that, in correlated systems, bandwidth modulation can induce an abrupt change in orbital hybridization rather than a rigid response to interfacial strain.

**Supplementary Figure 9.** Comparing the role of orientation design and film thickness in engineering the IMT property of VO_2_. As the film thickness increases, the interfacial strain gradually relaxes, resulting in a gradual elevation of *T*_IMT_ toward the bulk value of ~341 K, owing to the strain relaxation. However, the magnitude of this thickness‑induced *T*_IMT_ variation is considerably smaller than that induced by the crystallographic orientation design.

**Supplementary Figure 10.** The *ρ*-*T* curves as compared for VO_2_/TiO_2_ (102) with different thickness. In addition, 20 nm-thick VO_2_ film deposited on TiO_2_ (102) substrate displays similar *T*_IMT_.

**Supplementary Figure 11.** Mirror symmetry for VO_2_ grown on low-Miller-index **a,** TiO_2_ (001) and **b,** TiO_2_ (100) substrates. The VO_2_ films deposited on the TiO_2_ (001) and TiO_2_ (100) substrates exhibits two mirror planes, contrasting sharply with the counterpart grown on the (102)-faceted TiO_2_ substrate, which lacks any mirror planes.

**Supplementary Figure 12.** The *ρ*-*T* curves as compared for VO_2_/TiO_2_ (100) heterostructure along different *in-plane* directions. Nevertheless, no pronounced anisotropy in *in-plane* electrical transports is detected for the VO_2_ film deposited on low-Miller-index (100) crystallographic orientation.

**Supplementary Figure 13.** Schematic illustration of hydrogenation in the strongly correlated VO_2_ system via the hydrogen spillover method. The process begins with dot-shaped Pt catalysts sputtered onto the VO_2_/TiO_2_ heterostructure, which reduce the energy barrier for H_2_ dissociation from an H_2_/Ar gas mixture.^[2-4]^ At elevated temperatures, the resulting atomic hydrogen dissociates into protons and electrons at the triple-phase boundary, which then intercalate into the VO_2_ lattice, completing the hydrogen evolution.

**Supplementary Figure 14.** Comparison of hydrogenation-related resistance modulation (*R*_H_/*R*_0_) between (102)_R_-faceted and (100)_R_-faceted VO_2_ films. Under identical hydrogenation conditions, the (102)_R_-faceted VO_2_ film exhibits a greater resistance modulation, in comparison with (100)_R_-oriented counterpart, indicating that deliberate engineering of the *c*_R_-axis orientation can accelerate proton diffusion.

**Supplementary Figure 15.** Thermal activation energy (*E*_a_) for **a**, VO_2_/TiO_2_ (100) heterostructures, **b**, hydrogenated VO_2_/TiO_2_ (100) heterostructures **c,** VO_2_/TiO_2_ (102) heterostructures and **d,** hydrogenated VO_2_/TiO_2_ (102) heterostructures via linearly fitting their ln(*ρ*)-1000/*T* tendencies. **e,** Comparison of the hydrogenation-induced change in *E*_a_ for VO_2_ films grown on different crystallographic orientations. Protonation leaves the *E*_a_ of (100)-faceted VO_2_ largely unaffected. In marked contrast, it induces a two-fold increase in the *E*_a_ of the (102)-faceted counterpart. These findings constitute direct evidence that protonation kinetics can be accelerated in the VO_2_/TiO_2_ system via the artificial design of crystallographic orientation.

**Supplementary Figure 16.** Three-dimensional **a**, H **b**, V and **c**, Ti element maps for hydrogenated VO_2_/TiO_2_ (100) heterostructure as characterized by using the time-of-flight secondary ion mass spectrometry (ToF-SIMS) analysis. According to the ToF-SIMS result,^[5]^ the identical element distribution as observed for V and H elements demonstrates the effective incorporation of hydrogens into the lattice of VO_2_ film deposited on the TiO_2_ (100) substrate upon hydrogenated.

**Supplementary Figure 17.** Depth profile of the hydrogen, titanium and vanadium elementary distribution within the lattice of VO_2_ film deposited on the TiO_2_ (100) substrate as characterized by using time-of-flight secondary ion mass spectrometry (ToF-SIMS) technique. The H signal in the VO_2_ film significantly exceeds that of TiO_2_ substrate, demonstrating an effective hydrogen incorporation.

**Supplementary Figure 18.** *ρ*-*T* as measured for VO_2_/TiO_2_ (100) and VO_2_/ TiO_2_ (102) heterostructures under **a,** hydrogenated and **b,** dehydrogenated. A greater resistivity change was observed for the VO_2_/TiO_2_ (102) heterostructure compared to the (100)-oriented VO_2_ under identical hydrogenation conditions. This difference is attributed to the tilted oxygen channels realized in the (102)-oriented film, which facilitates hydrogen ion intercalation. After the same duration of air exposure, the sharpness and the phase transition temperature was gradually recovered for VO_2_/TiO_2_ (102), while the (100)-oriented heterostructure remained in the hydrogenated insulating phase.

**Supplementary Figure 19.** Full spectra for soft X-ray absorption spectroscopy (sXAS) of VO_2_/TiO_2_ heterostructure through hydrogenation.

**Supplementary References**

[1] X. Zhou, X. Yao, W. Lu, J. Guo, J. Ji, L. Lang, G. Zhou, C. Yao, X. Qiao, H. Ji, Z. Yuan, X. Xu, Manipulating the Hydrogen-Associated Insulator-Metal Transition Through Artificial Microstructure Engineering. *Adv. Sci.* **2026**, 13, e10771.

[2] X. Zhou, H. Li, Y. Jiao, G. Zhou, H. Ji, Y. Jiang, X. Xu, Hydrogen‐Associated Multiple Electronic Phase Transitions for d‐Orbital Transitional Metal Oxides: Progress, Application, and Beyond. *Adv. Funct. Mater.* **2024**, 34, 2316536.

[3] X. Deng, Y.-X. Liu, Z.-Z. Yang, Y.-F. Zhao, Y.-T. Xu, M.-Y. Fu, Y. Shen, K. Qu, Z. Guan, W.-Y. Tong, Y.-Y. Zhang, B.-B. Chen, N. Zhong, P.-H. Xiang, C.-G. Duan, Spatial evolution of the proton-coupled Mott transition in correlated oxides for neuromorphic computing. *Sci. Adv.* **2024**, 10, eadk9928.

[4] W. Karim, C. Spreafico, A. Kleibert, J. Gobrecht, J. VandeVondele, Y. Ekinci, J. A. van Bokhoven, Catalyst support effects on hydrogen spillover. *Nature* **2017**, 541, 68.

[5] P. P. Balakrishnan, D. Ferenc Segedin, L. E. Chow, P. Quarterman, S. Muramoto, M. Surendran, R. K. Patel, H. LaBollita, G. A. Pan, Q. Song, Y. Zhang, I. El Baggari, K. Jagadish, Y.-T. Shao, B. H. Goodge, L. F. Kourkoutis, S. Middey, A. S. Botana, J. Ravichandran, A. Ariando, J. A. Mundy, A. J. Grutter, Extensive hydrogen incorporation is not necessary for superconductivity in topotactically reduced nickelates. *Nat. Commun.* **2024**, 15, 7387.
